# Algorithmic emergence? Epistemic in/justice in AI-directed transformations of healthcare

**DOI:** 10.3389/fsoc.2025.1520810

**Published:** 2025-02-07

**Authors:** Imo Emah, SJ Bennett

**Affiliations:** ^1^Faculty of Health, Social Care and Medicine, School of Medicine, Edge Hill University, Ormskirk, United Kingdom; ^2^Department of Geography, Lower Mountjoy, Durham University, Durham, United Kingdom

**Keywords:** digital health, medical AI, data justice, epistemic injustice, emergence

## Abstract

Moves toward integration of Artificial Intelligence (AI), particularly deep learning and generative AI-based technologies, into the domains of healthcare and public health have recently intensified, with a growing body of literature tackling the ethico-political implications of this. This paper considers the interwoven epistemic, sociopolitical and technical ramifications of healthcare-AI entanglements, examining how AI materialities shape emergence of particular modes of healthcare organization, governance and roles, and reflecting on how to embed participatory engagement within these entanglements. We discuss the implications of socio-technical entanglements between AI and Evidence-Based Medicine (EBM) for equitable development and governance of health AI. AI applications invariably center on the domains of medical knowledge and practice that are amenable to computational workings. This, in turn, intensifies the prioritization of these medical domains and furthers the assumptions which support the development of AI, a move which decontextualizes the qualitative nuances and complexities of healthcare while simultaneously advancing infrastructure to support these medical domains. We sketch the material and ideological reconfiguration of healthcare which is being shaped by the move toward embedding health AI assemblages in real-world contexts. We then consider the implications of this, how AI might be best employed in healthcare, and how to tackle the algorithmic injustices which become reproduced within health AI assemblages.

## 1 Introduction

Digital transformation of the health service is being sold as a silver bullet to fill both funding and knowledge gaps. Increasingly this vision centers upon Artificial Intelligence (AI) models, particularly deep learning which leverages neural network-based pattern recognition from data; in this paper we are primarily referring to deep learning when we use the term AI. Proponents argue that this “deep medicine”—a form of health AI assemblage where deep learning is used to facilitate several medical diagnostic and treatment processes (Topol, 2019a)—can combat the inefficiencies of clinical interactions, augmenting them with deep learning models which can integrate various sources of data, across medical specialties and even outside the clinic from patients' data shared by external platforms and databases such as digital health applications or social care services (Topol, 2019a). The promise is that AI medicine can tackle limitations of current medicine, especially in thorny domains such as Mental Health and chronic pain diagnosis and treatment. Modeling emergent properties of datasets is seen to escape human limitations in pattern recognition and information processing (Topol, 2019b). To fully understand the emergence and potential impact of these sensibilities, it is valuable to examine the narratives and histories that have shaped them. Medical AI is framed, developed and applied within the cultural context of the Evidence Based Medicine (EBM) paradigm, with this entanglement shaping both development and interpretation of model outputs. In this paper, we highlight processes of datafication, algorithmization and automation (Ricaurte, 2022) within medical AI emergence, processes which facilitate [Western] cultural and political domination, supported by taken-for-granted notions of improved evidence-based practice. There is a risk that rather than truly presenting an alternative to the limitations of current approaches, these methods concretize existing problems, simultaneously recreating recursive social looping effects and obscuring them with slick, simple mathematical outputs (Beer, 2022). We contribute two main observations via a conceptual analysis of the interactions between EBM and AI in the UK healthcare system and discuss how systemic emergence might be shaped by the evolutionary pressures posed by the requirements of AI systems. We highlight processes of algorithmic emergence, drawing from literature on how AI models produce exemplars from data, and examining entanglements of these models with higher level systemic emergence of healthcare organization. Building on this analysis, we reflect on implications for political governance of medical AI, particularly algorithmic fairness and data justice.

We employ the concepts of emergence and emergent properties as tools to illustrate and help interrogate the ways in which AI can pose a challenge for patient engagement in healthcare design and provision, and even restructure the healthcare system within which they are employed. The notion of emergence—more specifically, of “emergent properties” (O'Connor, 2015) describes the characteristics of a system produced by interactions of multiple agents which are difficult or impossible to predict from understanding the properties of the individual agents. Although emergence as a named topic only arose within the last few decades, the study of complexity and self-organization of simple, low-level units into complex systems has a long history, and includes the study of technological systems as fundamentally emergent (Johnson, 2002). Esayas (2017) describes emergence as employed across numerous domains and disciplines, and alternately applied as a concept, theory and methodology in research. Johnson (2002) surmises the concept as “structure that you would not necessarily predict from the rules” (p. 90). Much like with a beehive, where a focus on the actions of each bee obscures the functioning of the hive, we argue that the focus on developing better machine learning models or computational processes used in healthcare potentially overshadows the systemic changes produced by integration of such technologies. Continuing to treat the individual parts of the system contributing to and affected by such emergence without addressing the changes driven by interactions of the parts can have negative implications. For this reason, we avoid the common analytic stance which seeks to identify a “root cause” or key driver within a system, and instead consider how interactivity and co-constitution enable a better understanding of the behaviors and effects of the system (Johnson, 2002). We focus on the UK health system to understand the role of emergence in the configuration of current practices, and we maintain this focus in deliberations of the potential impact of medical AI.

## 2 AI and medicine

According to a recent report by the Alan Turing Institute, one in four UK doctors is employing AI in some way, in the wake of the release of popular large language models including ChatGPT (Hashem et al., 2024). Prominent discussions of health AI suggest that it can tackle the shortcomings and challenges faced by dominant models of healthcare. Indeed, surveys of deep AI literature suggest that it is most frequently applied to tackle cases with high degrees of uncertainty and complexity in order to improve efficiencies (Piccialli et al., 2021). Quite often these promises, and responding discussions, focus on the roles of AI models, individual or institutional acceptability and equitable access, and perhaps how these are linked to availability of information systems and large and/or representative datasets (Horgan et al., 2020; Roppelt et al., 2023; Zahlan et al., 2023). In practice, however, AI interventions often fail to be useful in clinical contexts (Roberts et al., 2021). Despite this, the introduction of their modes of knowing and thinking seem to be endemic despite the lack of evidence for their usefulness. Instead of offering improvement to existing paradigms (and derived processes) of medicine, medical AI represents transformation into a new, emergent paradigm rooted in drives toward innovation filtered through existing structures and sensibilities (Essén and Lindblad, 2013). Understanding the potentials for this emergence, and its corresponding impacts, requires a critical examination of some of its interacting components, including medical data, machine learning models, the health service, and the features (and systematic effects) of the knowledge paradigms which shape current medical practices as rooted in Evidence-based Medicine.

### 2.1 Ecologies of data

AI data comes in numerous forms, often domain-dependent although there is a sustained effort toward multi-modal models which combine data from numerous sources. This data includes natural language in healthcare literature, Electronic Health Records, clinical notes, images including MRIs, CT scans, x-rays, and structured datasets such as excel spreadsheets and SQL databases. This use of multiple, massive data sources operates within a broader narrative positioning data as an exploitable resource, spurring desire for greater access to data to assist the development of these models (Elish and Boyd, 2018). This also necessitates development and appropriation of infrastructures for collection and collation, with many of these enterprises leveraging geopolitical and economic inequities to outsource much of this “data work” to developing countries (Chandhiramowuli et al., 2024; Couldry and Mejias, 2019). In this way, applications of AI in healthcare appropriate and expand the “data gaze” - the ideals that shape the ways in which data is gathered, stored and modeled, and particularly, how data is used in political governance (Beer, 2018). This data gaze exerts the vision of AI representing an emergent process of “discovering” new associations and hypotheses, extending the capabilities of the current EBM paradigm which we discuss in more detail in the following sections. The data itself requires careful curation and transformation into a format amenable to AI models. To do so, medical AI systems are often dependent on data labeling practices, processes of knowledge capture which aim to model the “current state of medical knowledge and the tacit knowledge of medical professionals” (Zając et al., 2023, p. 357). These practices are often complicated and segmented, requiring the transformation of large amounts of information and various forms of input by non-specialist data workers (Chandhiramowuli et al., 2024) and *ad-hoc* workarounds by data curators (Thomer et al., 2022), yet these outputs must be deemed useful for the specific aims of the practitioners creating and utilizing medical AI (Bennett et al., 2025). These data journeys, the processes of selection and curation of data, are seen as sufficient to ensure appropriate model outputs which are relevant to context (Leonelli and Tempini, 2020), particularly when such outputs are translated through the EBM paradigm, as we examine further in our discussion of the shape and legacy of EBM.

### 2.2 The emergent properties of AI models

AI practice and models have emergent properties and are fundamentally shaped by uncertainty and ambiguity (Bennett, 2023; Grote and Berens, 2023). These characteristics are embedded by design, with the move to data-directed models where “discovery of a structure and its interpretation are inextricably entangled with what the researcher is looking for and the standards and expectations she sets, even when these remain implicit” (Campolo and Schwerzmann, 2023; p. 8). An attractive aspect of dominant AI approaches such as deep learning is their potential to extract meaningful patterns and relationships from data without requiring precisely described features and theoretical frameworks (Alom et al., 2019). Such seemingly serendipitous narratives of AI use terms suggestive of algorithmic emergence, where novel and complex discoveries are produced by applying relatively simple rules from a learning algorithm to large datasets, yet typically fail to acknowledge the intentional structuring of resources and processes which defines this emergence. For instance, as mentioned in the previous section, data itself is selected and made available through complex and often unacknowledged curation processes (Thomer et al., 2022), and algorithms are often selected to fit the capacities of the systems on which they would be applied, such as available computing power (Uddin et al., 2015). Applied within the healthcare context, medical AI risks becoming a “conjectural science” (Stark, 2023; p. 35), engaging in abductive, retrospective interpretation of outputs which is marketed as objective and representative of a broader truth. This is a distinct epistemological model from “empirical science”, which is characterized by reproducibility and consistency, and testing of hypotheses (Stark, 2023, p. 38), typified in current medical practice by Evidence-Based Medicine (EBM) which we discuss further in the following section. Deep learning and other similar AI methods reproduce conjectural science at scale, “automating conjecture” (Stark, 2023, p. 42) via Big Data and vast computational resources, where these are conflated with empirical science - associations in data treated as indicative of some underlying process or ground truth and requiring no further confirmation. This conjectural science approach focuses upon the ability to “identify” and model relations in datasets which are taken to indicate underlying processes and previously overlooked realities, though this process of “identification” is contingent on the nature of biases in the dataset. Campolo and Schwerzmann (2023) point out that these forms of AI herald a shift from a rules-based approach (where specific rules must be abided by) to a data-driven approach, where parameters describing the phenomena that AI algorithms are attempting to model are extracted from the training data and employed as “exemplary representations” for ongoing work (p. 2). This shift from rules to examples is not just a description of how AI models work but describes governance within the systems in which these models are applied. The emergent properties of deep AI methods form their most useful features, but their contingent and conjectural nature must be accounted for in developing and engaging with medical AI. In effect, AI moves medical research from explanatory to predictive, from clearly defined goals and inputs to emergent processes driven by data, though data used to train predictive models are invariably shaped by the normative understandings of the model developers. The integration of the emergent identifications of AI models within the processes and practices derived from EBM thus presents a move to a different modality of medicine, one granted legitimacy from the conflation of the premises of both approaches, producing an amalgam with the potential to radically change experiences of healthcare, particularly for those already marginalized by existing knowledge and care practices.

## 3 EBM and the emergence of the UK health system

The general practice of Evidence-based Medicine (EBM) as “the conscientious and judicious use of current best evidence from clinical care research in the management of individual patients” (Sackett et al., 1996, p .71) has a long history. The adoption of this perspective sits within a larger move toward using statistics and quantification to direct governance and evidence productivity (Rose, 1991), translated through socio-political and administrative processes within the care context. EBM has underpinned health-related practices in the UK healthcare system since the 1950s, replacing the somewhat eclectic array of more practitioner-directed approaches to healthcare (Benech et al., 1996; Ratnani et al., 2023). The UK National Health Service (NHS) is typically framed as a centralized, top-down system, all the way from its birth in 1948 in the wake of the second world war. However, it is perhaps better conceptualized as resulting from several periods of “manipulated emergence” (Harrison and Wood, 1999) the repeated prompting, absorption and reconfiguration of multiple self-organizing systems that were created in response to sociopolitical pressures, dominant health concerns and available resources of their local context (Klein, 1983). Within this interplay of forces, EBM often served an agenda to provide information on the service effectiveness and efficiency, reflecting neoliberal ideals that proliferated in the commercial sector which was primarily concerned with improving competitive advantage and post-war economic recovery (Bayliss, 2022; Sturgeon, 2014). For instance, the multiple reforms to the NHS—which, from its inception, augmented, amended and structured the work of different healthcare providers—demonstrated moves between closely monitored administrative control and regionally variable or marketized devolution of governance and responsibility, usually reflecting the ideological and accountability positions of the State (Hunt, 2013; Devlin and Lunn, 1986; Joyce, 2001; Klein, 1982). These moves were augmented by developing technologies that improved availability and access to computing power and digital information collection systems, producing a service structure that promoted the documentation and categorization of service users for effective administration. More recently, the establishment of Integrated Care Systems within the Health and Care Act (2022) was a centralized adoption of several local initiatives created to address the complex health and social care needs of service users through joint interprofessional working arrangements, disrupting the previous separated configurations of these healthcare domains which had been so designed to ease administration and funding processes (Harrison, 2022). This legislative endorsement of collaborative health and social care provision was intended to help to improve care pathways and reduce expenses in time and resources, both of which align with governmental ambitions on maximizing service efficiency, helps extend its administrative reaches while embedding contemporary concerns (Guy and McHale, 2023).

This perspective of manipulated emergence helps highlight the institutional power structures needed to craft and maintain the health system, and “evidence-based medicine/practice” has been invoked as the basis of ideologies and processes that consolidate and legitimize organizational activities (Harrison and Checkland, 2009). In line with desires to streamline administration, optimize healthcare provision and demonstrate value for money, available evidence has been used to guide development and (re)structuring of the health system, and has shaped the features and priorities of the service, including the development of health research initiatives/organizations, digital technologies and data-collection processes and infrastructure such as Electronic Health Record systems (EHRs). The dominant application of the EBM paradigm has also shaped the knowledge perspectives and practices of health professionals, often through emphasizing a reliance on measurable data over subjective knowledge (whether of the professional or patient) in shaping clinical decision-making, a feature which holds implications both in the current healthcare landscape and for the expansive use of such data in automated processes such as AI. At the same time, pushback against the population-centered objectivist view popularized by EBM spurred concerns around normative conceptualizations of patients and the development of care practices around these norms (Greenhalgh et al., 2015). These concerns, bolstered by patients' customer-centered expectations from experiences of other public-facing largely commercial services, spurred initiatives which focused on the adaptation of healthcare to suit the context and concerns of the patient: ideals of person/patient-centered care (Engle et al., 2021; Sturgeon, 2014; Warden, 1987). Such capacity to personalize healthcare has often been promoted as a benefit of AI applications, which are supposed to use patient data to tailor diagnoses or decisions with greater degrees of accuracy (Topol, 2019a), though the underlying processes of abstraction and data modeling used for these tailored outputs are typically obfuscated. To better understand some of the implications for the move to AI-assisted medicine, the next sections will explore the paradigm of EBM to highlight how its features shape the development and potential impacts of AI integration within the health system, with a notable focus on health inequities and justice.

### 3.1 An overview of the EBM paradigm

This section lays out some key ideas and critiques of evidence-based medicine (EBM), which shape both the delivery of healthcare and production of its inequities. As foregrounded earlier, the dominant adoption of the EBM approach emerged in response to concerns around equitable standards of care, improving efficiencies within the health system, and the provision of reliable (often expected to be quantifiable) information to guide clinical decision-making, especially given the potentially fatal consequences of such decisions (Benech et al., 1996. These led to a proliferation of statistical methods as the cornerstone of health-related research to support the development of diagnostic and treatment guidelines. Broadly speaking, such methods use associations between data measures to support hypotheses on cause and effect in relation to health issues, associations predicated on certain assumptions about the data and the populations from which they are derived (such as normal distributions of traits, or probabilistic sampling). While the philosophical basis of EBM has been argued to support a range of forms of evidence and to broadly represent an epistemological view to optimizing clinical practice (Djulbegovic et al., 2009), actual knowledge practices are more contextually contingent and socially constructed, though these dimensions are often obscured by value-neutral representations of the approach.

These contextual and social processes which have shaped the real-world applications of EBM are typified in Miranda Fricker's concept of Epistemic Injustice; this arises when the knowledge contributions of certain people are diminished due to facets of their identity, a distinct type of injustice where someone is wronged specifically in their capacity as a knower (Fricker, 2007), including whether there are philosophical concepts which individuals can employ to communicate their knowledge. The processes through which “evidence” is created—though posited as “objective” if it follows certain rigorous research principles—are shaped by the features of knowledge generators, including researchers, journal editors, distributors, etc. who are involved in the framing of issues, selection of research methods, and dissemination of research findings to influence healthcare practice and policy decisions. In the UK context (which extends to global influence), these knowledge generators are largely from majority social groups and in positions of power and social privilege (White, heterosexual, middle class, English-speaking, able-bodied), and when leading investigations of issues affecting people from a range of dissimilar backgrounds, these processes can be impacted by often unacknowledged systemic racism, a “public health emergency” that produces inequities for minority groups (Ellis et al., 2021). Epistemic injustice pops up within all aspects of EBM processes resulting from “bias and distortions” that are sidelined by the focus on legitimizing results primarily through assessing the methods used, for instance the prioritization of research on conditions which can be investigated through clinical trials, the selection of comparators which can demonstrate significant differences, the use of outcomes which are measurable in short research timeframes, the exclusion of patient groups with characteristics which may muddy the data, even though these groups will still be affected by decisions made on the study results (Michaels, 2021). These distortions are especially concerning as a large body of research (especially around health technologies) is conducted and selectively disseminated by organizations with sufficient funding and commercial interests, and such selective evidence shapes how healthcare resources are distributed, and what benefits are accrued to which patients (Michaels, 2021, p. 420).

Beyond the contributions to significant changes in medical practices, the EBM approach reshaped understandings of health issues by influencing the ontological baseline of the health system itself. In particular, it emphasized a focus for care practices to be based on what was objectively knowable, and for findings to be statistically significant and generalizable - applicable to most or all members of a target population, an expectation which contributes to the erasure of diversity and devaluation of impacts on minority groups (Chapman, 2023). This focus naturally led to prioritizing, and granting authority to, knowledge created through experimental and/or population-based studies, typically with a focus on singular issues where significant associations could be demonstrated. Thus, EBM not only informs how biomedical knowledge should be used in practice, but shapes what even counts as relevant or appropriate knowledge. For instance, the National Institute of Health and Care Excellence (NICE) which oversees the standards for care in the UK relies heavily on the demonstration of such evidence for the adoption, alteration or abandonment of practice guidelines, processes shaped and constrained by the very procedures which lead to availability of such evidence. A well-worn example of this can be seen in pharmaceutical guidelines on drug dosages, where evidence for efficacy of medication was drawn largely from male individuals (who were in the position to consent to, and participate in such trials) and applied to the population, producing issues and often unrecognizable complications for those whose physiology differed from this trial population in a range of ways (Perez, 2019). Similarly, the understanding of systematic reviews as the highest levels of evidence for decision-making (Goldenberg, 2009) has often been transformed from a quality ascribed to investigative processes (based on considerations of potential sources of biases and inaccuracies) to a necessary condition for acceptable knowledge or to justify any such health-related decisions. This move often systematically disenfranchises minority populations, as the availability of studies like randomized control trials on these populations tend to be unavailable or inadequate, and available research typically shows significant heterogeneity to be disqualified from meta-analyses (Higgins et al., 2002; Hussain-Gambles et al., 2004). This selective application of evidence can be used to warrant withholding care, for instance the NHS-commissioned ‘Cass Review' into children's gender services cited insufficient evidence from randomized control trials to justify discontinuing provision of these services (Cass, 2024). In doing so, the report disregarded the difficulty and ethical constraints of designing and implementing randomized control trials for children in general, let alone a minority group, and the unsuitability of RCTs for studying these services (Horton, 2024).

Critiques of EBM range from its ontological to technical and sociological dimensions (Mullen and Streiner, 2006); for instance, the assumption that data-driven associations produce a clearer or more “objective” understanding of diseases or patient groups has promoted an enterprise for enhanced collection/uses of data, basing precision in more elaborate levels of abstraction. Procedurally, the insistence on statistical significance (along with pressures to show results from research endeavors) contributed to the phenomenon of “p-hacking”, where data and analytical methods are selected to enable the production of significant results (evidenced through the p-value which indicated the probability of chance associations between measured data (Adda et al., 2020). Similarly, the requirement of large sample sizes and population-based studies assumes similarity or ameliorable differences between constituent individuals, against which statistical associations can be ascribed to a handful of measured properties which have not been “controlled”. Furthering this, as through the establishment of clinical guidelines, EBM has influenced the organization and delivery of healthcare itself, emphasizing scalability and standardization across care contexts (Ost et al., 2020; Teig et al., 2023). These emphases have been argued to produce a mode of governmentality and governance predicated upon individualist assumptions despite (and obfuscated by) evidence being derived from analysis of large datasets, potentially devaluing discrete individuals receiving care and amplifying existing societal inequities (Greenhalgh et al., 2015). The categorization practices needed to render data intelligible for analytic processes often become stand-ins for properties of the individuals themselves, and associations made to these categories become individualized in the care context; an example being the use of ethnic categories to compare risk of disease, from which any observed differences then become applied to individuals of that ethnicity as through “risk scores”. These issues are often described as limitations of the data or research methods, obscuring the power dimensions and sociological biases inherent in epistemic processes and institutional practices (Heggen and Berg, 2021). In this way, the epistemic injustices produced in the creation and uses of “evidence” are erased or framed as inevitable, and the impacts of these injustices are stripped of significance - especially as those affected are disempowered.

The issues discussed in this section reveal some of the qualitative subtleties and the influences of power and privilege in shaping how the premise of “evidence-based medicine” is translated in practice, using data that is often curated to serve the understandings or interests of investigators, which holds implications for what (and whom) can be understood through such data. This, combined with concerns about innovations to healthcare practice and perceptions that data associations hold the key to improved understandings of health, have contributed to an inheritance of ideals and tools that paved the way for AI integration, which we will discuss in the next sections.

### 3.2 The legacy of EBM in AI for healthcare

The centrality of EBM to current healthcare practices underscores an approach to health-related considerations and administration; decisions made about individuals receiving care are often based on how they fit within expectations derived from a population, emphasizing a dominantly normative paradigm that shapes health professionals' understandings of, and responses to, patients. This ontological and epistemic shift promotes a flattening of individuated nuances and lends to expectations for health data to facilitate decision-making through the mapping of such data along calculable indices. Studies within the sociology of health, illness and diagnosis have examined the role which tools and measures play in framing, legitimizing and attempting to standardize disease and diagnostics, not without pushback from clinical practitioners (Jutel, 2009; Nelson, 2019; Nettleton, 2020). The Body Mass Index (BMI) measure provides an example case for this, providing a convenient, simple and easily calculable measure which has been integral in solidifying the factuality of obesity as an “epidemic”, reifying the disease concept by providing a means by which to evidence it - presenting it as an objective measurable quantity despite its complex socio-political origins (Gutin, 2018; p. 2). In this case, statistical tools came together with social sensibilities to create a new epidemic. The EBM framing of seeking to find causality can alienate patients and result in epistemic injustice, particularly around patients' participatory involvement in the creation and contextualization of health-related knowledge and practices, due to the prioritization of data, theories and concepts deemed relevant and endorsed by health practitioners and administrators (Goldstein, 2024). The data-as-authoritative epistemic lens provides the baseline to support for AI integration in clinical practice, and common discussions of AI applications suggest a vision to recreate or facilitate this mode of EBM governmentality in healthcare. AI often draws on data that already exists, in the clinical context being EBM studies, EHRS, imaging etc, but it also shapes and directs EBM based studies and future research, whether through hypotheses built on emerging data associations or studies which make use of technologies.

The move toward AI-driven medicine has the potential to fundamentally re-organize care relationships and possibility spaces for data justice within health and medicine. At the most obvious level, this is evidenced by the effect of AI hype on recruitment, training and retention of doctors in the specialisms which AI models claim to tackle (Byju, 2024). Medical AI approaches such as deep learning concurrently shift the burden of responsibility to the patient in pinpointing ‘individualized’ lifestyle and genetic features (Baumgartner, 2021), and limit patient agency by removing ‘subjective’ elements from the equation, taking power from testimony and self-report and relying on modeling relationships between groups deemed similar to the patient at hand. For one, it neglects the socio-materially negotiated nature of EBM. This has already proven problematic in practice; for example, given evidence that clinicians tailor EBM to their own needs. The team working on the failed IBM Watson system, a project which set out to predict cancer using deep AI, sought to understand why it did not succeed; their investigation revealed that physicians often utilize information outside the primary point of the study at hand, and adapt their response in a way which is qualitatively obvious but concealed when considered based on data alone (Ross and Swetlitz, 2018). This example highlights a broader trend/issue where AI applications can practically serve to distance clinicians from interpretation of literature and other sources; this can be done for a range of reasons including increasing efficiencies, standardizing care, freeing up practitioners' time for other issues, and on. This is further complicated by the emergent nature of deep AI techniques whose outputs are translated through an EBM lens, especially as the processes and exemplars used to develop the AI outputs are typically obscured and thus shielded from critique. The acceptance of such outputs can produce a sort of automated ecological fallacy, where “personalized” findings are produced from associations taken from group-level data, with the belief that models created from such data can be applied at the individual level as though all members of the dataset are interchangeable (which is an extension or exercise of the ideal of “generalizability” of findings where the dataset is considered representative of the population).

This implicit bias toward group-level interchangeability can be seen in moves to create “synthetic data” to advance uses of AI in contexts where primary data is poor or missing, emphasizing the value of such abstractions over the participation of underserved communities to ground understandings. These moves toward synthesizing data for modeling have largely been supported by Western organizations for use in lower income “data poor” countries (Milan and Treré, 2020), with the perceptions of similarities between countries used to justify the uses of such data to understand and make decisions about their populations. Ricaurte (2022) discusses such uses of AI in the current Western-dominated largely capitalist sociopolitical landscape as capable of producing real and symbolic violence through three epistemic processes (which have parallels within current EBM practices): datafication, algorithmization and automation. Datafication, the conversion of people and phenomena into categories and quantitative operations, leads to qualifications of the world in ways that often serve hegemonic interests and aids the reproduction of hierarchies of value; for instance, utility measures like quality-adjusted life years (QALYs) which are used to guide decisions in health economics inherently devalue the lives of disabled people (Williams, 1996). Following this extractive transformation into data, algorithmization presents the structures, relationships and processes of people and societies as calculable models, with the aim to convert various kinds of input into outputs - usually predictions. Despite how they are often presented, these algorithms are not simply products of intricate computing processes, but are imposed through influential (often colonial) structures of knowledge production where existing biases and inequalities are often encoded into norms (Arora et al., 2023). However, the narratives of unbiased algorithmic outputs from AI favor moves toward automation, where interactive and administrative processes are increasingly removed from human intervention. Such moves will reproduce encoded biases in decision-making, foreclosing possibilities for accountability and responsibility.

Taken together, the integration of AI within the current landscape of evidence-based practices and the priorities of healthcare governance opens questions of who is served or dispossessed by the growing applications of algorithms and the promotion of data-dominant discourses which predominantly concretize the perspectives and logics of existing power structures (Ricaurte, 2022). Unlike popular deliberations of how AI can best be embedded within the existing health system, we use the perspective of emergence to anticipate how AI algorithms could more fundamentally reconfigure healthcare through interactions with human, material and sociopolitical elements. For one, as mentioned earlier, current and proposed applications of AI in healthcare (and the healthcare sector more generally) are governed through capitalist sensibilities with dominant interests in productivity and cost-efficiency (Abel-Smith, 1992; Sturgeon, 2014). Indeed, understandings of health itself are shaped by these sensibilities, for example, how neurodiversity is only deemed appreciable if it impacts on economic productivity which has been framed as central to a meaningful existence (Chapman and Carel, 2022). Such perspectives have also proliferated through historical (and ongoing) colonialist expansions and globalization, pushing the dominant interests and ideals of largely White Western societies at the cost to situated local knowledge and interests (Ricaurte, 2022), a dynamic which would drive (and be reflected in) applications of AI, though successful implementation would be based on available institutional, economic and computing power. Combined with understandings of AI outputs as data-derived - mirroring an epistemic lens developed through EBM, which has concretized ideas on what knowledge, issues and populations are deemed significant - and bolstered by concerns about workforce stability, the positioning of AI to assist healthcare would more likely result in promoting automation across as many domains as possible. Furthering automation (and its supportive logics and infrastructure) could contribute to re-conceptualizations of patient groups (by care professionals, administrators/governing bodies, or patients themselves), and promote refinements of care practices to fit such automated processes and realize the efficiencies of the system, with any resulting harms concentrated on epistemically and socio-economically marginalized groups. To explore the implications of this, we consider some of the effects of AI integration on the knowledge paradigms and processes within current healthcare practices, including issues of data justice, equity and governance.

## 4 Discussion

In previous sections, we hoped to highlight the interacting elements that contribute to algorithmic emergence within the UK health healthcare system. Nestled within this systemic algorithmic emergence is the issue of emergence within AI models themselves, as techniques which are open-ended, shaped by data, contingency and practices of norm construction. We see algorithmic emergence as at once the study of the contingent ways in which various socio-material factors come together with AI algorithms to shape the healthcare system, and a feature inherent to AI modeling. The contingent factors which have shaped the existing healthcare system will shape the ways in which emergent AI models are interpreted. AI, as such, is more than just another tool within the inventory of healthcare practitioners. Rather, both the material and epistemic requirements of AI serves as a kind of evolutionary pressure that could fundamentally shift the understandings and acceptable outcomes of healthcare systems, boosted by the legitimization of the capacity for AI to more accurately model reality. This pressure is exerted in the promotion of socio-material interactions creating the conditions for AI proliferation and reshaping conceptualizations of healthcare, and in the stripping/devaluation of qualitative nuance. AI in general benefits from positive beliefs around the offerings of big data, and this certainly feeds into optimism around deep medicine. In particular, deep medicine benefits from hybridized epistemic framings with EBM. This can be seen in framings which praise “their ability to build off of existing knowledge to predict new knowledge” (Arnold and Tilton, 2020, p. 310). As discussed in this paper, much of UK medical training and research is based on the paradigm of Evidence-Based Medicine (EBM), driven by testing and refuting hypotheses based on large amounts of data to produce inferences which are deemed applicable to naturally variable populations. AI models, by contrast, are framed to offer a more personalized model which can leverage a far greater range of data sources and automated methods (Rennie, 2024), posited as a data-driven approach rather than the hypothesis driven one of EBM. Here we focus on a few specific implications of this hybrid of EBM and AI; ecological shaping and epistemic mismatches, which we discuss further in the next section.

### 4.1 Implications for integration of AI in medicine

AI's portrayal of simplified data-derived linear outputs are concurrently defined by complexity and emergence, “in every singular action of an apparently autonomous system, then, resides a multiplicity of human and algorithmic judgements, assumptions, thresholds, and probabilities” (Amoore, 2020, p. 64). Furthermore, they can embody limitations such as circular reasoning where existing assumptions and studies are used to define the AI problem space, producing automated conjectures based on the dataset and features used to create the model, “taking marginal and irrelevant details as revealing clues” (Stark, 2023, p. 36). Common framings of AI as data-driven rather than hypothesis-driven neglects the complexity introduced by these idiosyncrasies, and the impacts of domain-specific paradigms of interpretation. These models garnish users with singular outputs derived from complex, cross-domain sources in space, in a context assumed to be naturalist (Guersenzvaig, 2024). Medical AI represents a shift from reporting on existing populations, and tailoring medical advice based on the outcomes of these studies in the Evidence Based Medicine (EBM) approach which dominates the field, to creating new populations from interpolations of existing datasets with experimental variables (Jacobsen, 2023). In creating datasets from composite data points, and modeling relationships between categories to provide inferences, medical AI projects are enacting new categories of disease and patient, transforming a plurality of inputs into a composite object. These reconfigured amalgamations undercut current representations of the patient, disease and population, and risk supplanting various communities of practice, resulting in more limited epistemic agency and reasoning.

Fusing an Evidence Based Medicine (EBM) mindset with AI infrastructures and outputs obscures the presence of complexity and the implications of recursivity, both in social shaping of data and in the underlying logics of the methods, within AI systems. Furthermore, technology has the tendency to add complexity to the domains in which it is applied, rather than providing the simplifying force which is hoped for (Esayas, 2017). These applications of technologies are further shaped by the priorities of the domains within which they are employed - for instance, the dominant concern of healthcare organizations to demonstrate “value for money” in their care practices incentivises a focus on issues where such technologies can demonstrate their value. In a similar light, time constraints and the authoritative weight given to data-derived outputs lends to a further devaluation of patient consultation/testimony and a push toward basing disease-related knowledge and treatments on information constructed from alternative data sources. For instance, a large body of literature highlights the overuse of diagnostic testing to fast-track clinical decision-making (Müskens et al., 2022; Wallace and Fahey, 2018), and experiments on human-computer interactions suggest that deferrals to AI are more likely to occur in situations of ambiguity (Klingbeil et al., 2024). Coupled with time demands faced by health practitioners, pressures to meet performance targets and moves toward administrative streamlining that have historically shaped healthcare delivery, such changes have massive implications for care, potentially restructuring not only access to care, but also embedding systemic injustice.

### 4.2 Data justice and inclusion

Employing an emergent system such as current popular forms of AI like deep learning, with a command system (such as EBM) mindset is a particular concern given patterning of epistemic injustice at all levels of the medical system (Michaels, 2021). The forms of knowledge considered and granted authority as “evidence” are influenced on all levels by socio-political and power dynamics, shaping how issues (and populations) are conceptualized, how investigative processes are selected and implemented, and how knowledge is distributed and utilized. This contribution of knowledge goes beyond individual testimony, to contribution to a shared bank of knowledge even if the discrete information is not directly known by the individual. Epistemic injustice ameliorates and even disintegrates the agency of the patient “as knowers, interpreters, and providers of information” (Chapman and Carel, 2022, p. 1). Epistemic injustice is endemic in sites of knowledge production characterized by privilege and inequitable power dynamics including academia and medical practice (Rose and Kalathil, 2019). The structural racism which contributes to many health inequities (Rogers and Heard-Garris, 2023) does not stop at medical research design, Electronic Health Record keeping and AI model development. Shifting from population-mapping to modeling composite data relationships potentially severs clinicians and patients from shaping, querying and resisting these medical AI systems, and stifles moves toward their participatory involvement in reconfiguring healthcare through the increased valuation of clear-cut algorithmically-derived evidence. The narrative of employing AI methods to create a holistic view of the patient by feeding heterogeneous data sources to the model, framed as personalizing the model, has implications for governance which centers patient involvement and epistemic justice. This ‘holistic’ view is used to solidify a view of AI as providing objective outputs, with patient self-report being included in modeling processes which makes it appear that epistemic injustice is being tackled (Rath et al., 2024). In practice though, not only is there little to no transparency on how patients views are weighted, but as our examination of EBM has illustrated, patient knowledge is routinely downplayed in relation to other sources of data, and medical practitioner interpretation and valuation of patient reports are often dismissive and constitute a form of epistemic injustice (Heggen and Berg, 2021).

A growing body of literature has considered the potential for AI and related technologies to contribute to issues of bias, social inequities or other forms of injustice (Bennett et al., 2023; Hintz, 2024; Domínguez Hernández et al., 2024). Equity in these discussions of digital health tends to be focused on who has access to emerging technologies, often implying the socioeconomic benefits from such technologies. Likewise, bias tends to be focused around whether the user of said technologies will be discriminated against, obscuring the (de)valuation of ideals, knowledge and experiences which are central to technological development. Another important consideration is the socio-political infrastructures which AI both operates within and shapes the ongoing configuration of, and the interplay between participation, bias, and power. These factors limit who gets to contribute to (and benefit from) AI, and are often sidelined in discussions on how AI could be used “for good” in which standpoint of widespread or decontextualized positive value tacitly endorses the ideals and limited perspectives of those involved in technological proliferation (Moore, 2019). This problem of epistemic injustice in healthcare is of particularly concern when it comes to consideration of global medical practice and decolonization of medicine - epistemic injustice is far magnified when we consider global political divides (Bhakuni and Abimbola, 2021).

Quite often within AI, inequity is treated as a problem to be solved by introducing more data, neglecting the impact of the power structures within which data and models are collated and modeled. Concerns on the widespread uses of AI often center on the issue of another interpretation of “bias”, largely describing how the data on which the AI models are trained represent only a portion of the population and can lead to inaccuracies in the outputs; the natural antidote to these concerns are calls for greater representativeness or cautious interpretations of outputs (Miceli et al., 2022). Such discussions of bias also tend to present the issue as products of human error or insufficiency, with the assumption that accuracy can be achieved once an impartial computer system has access to the full range of data. Yet data itself is produced and utilized through complex ecosystems where multiple processes and decisions shape how a wide range of inputs are to be used, influenced by contextual power dynamics and socio-material factors (Miceli et al., 2020). The promise of AI to liberate healthcare from such issues can hardly be fulfilled given that the same power structures influence and often benefit from the systems and outputs of AI development. Despite the issues considered here, AI holds promise for meaningful contributions to healthcare, though its use must be considered with care and due regard for its mode of operation and the uncertain or ambiguous contexts it is employed within. For one, the access to high levels of computing power is valuable in generating ideas of association from information which would ordinarily exceed human capacities. However, enhanced abstraction of information from already limited systems should not replace the systematic changes required to enable equitable healthcare provision. Participation should be considered at all levels of knowledge generation and system design; this is an issue within the current paradigm of EBM, yet AI introduces a capacity for acceleration and automation of biases and limitations which poses a more significant challenge. Challenging these paradigms demands a conscious and critical examination of the taken-for-granted premises which shape current practices- for instance, how calls for participation can lead to tokenizing the viewpoints of minority participants (Táíwò, 2020), which is another manifestation of premise of interchangeability. A view to health AI assemblages which accepts and appropriately responds to lapses in understanding - while acknowledging power differentials and embedding participatory accountability structures - would better enable a conducive environment to ameliorate epistemic and algorithmic injustices.

### 4.3 Concluding remarks

The positioning and uses of AI in healthcare presents more than augmentations or alternatives to current practices, potentially prompting emergence of a different modality of healthcare which prioritizes particular forms of knowledge and reframes the recipients of care. In this article we presented a conceptual analysis of the interactions between Evidence Based Medicine and Artificial Intelligence in the evolving socio-political and technical context of the UK healthcare system, examining how this evolution might be shaped by the ideological and infrastructural demands of AI systems. Drawing on this analysis, we reflected on implications for political governance of medical AI, particularly algorithmic fairness and data justice, recognizing how interactions between the elements within healthcare provision can produce effects beyond the scope of typical process- or outcome-focused examinations of AI. More specifically, we highlighted how these shifts may present a challenge for patient autonomy, inclusivity and participation, and consequently impact on the possibilities for ethical and equitable healthcare provision. These hybrid EBM-AI epistemologies foreclose on certain futures and encourage moves toward others. While AI could facilitate some processes considered valuable in current healthcare arrangements, judicious uses of any such technology requires challenging dominant modes of conceptualizing, creating and employing evidence for guidance and governance, along with a careful and continuous re-examination of how (and to whom) harms are produced by healthcare reforms.

## References

[B1] Abel-Smith B. (1992). The Beveridge Report: its origins and outcomes. Int. Soc. Secur. Rev. 45, 5–16. 10.1111/j.1468-246X.1992.tb00900.x

[B2] Adda J. Decker C. Ottaviani M. (2020). P-hacking in clinical trials and how incentives shape the distribution of results across phases. Proc. Nat. Acad. Sci. 117, 13386–13392. 10.1073/pnas.1919906117 32487730 PMC7306753

[B3] Alom M. Z. Taha T. M. Yakopcic C. Westberg S. Sidike P. Nasrin M. S.. (2019). A state-of-the-art survey on deep learning theory and architectures. Electronics 8:292. 10.3390/electronics803029237716312

[B4] Amoore L. (2020). Cloud Ethics: Algorithms and the Attributes of Ourselves and Others. Durham, NC: Duke University Press.

[B5] Arnold T. Tilton L. (2020). “Depth in deep learning: knowledgeable, layered, and impenetrable,” in Deep Meditations: Thinking Space in Cinema and Digital Cultures, eds. K. Redrobe and J. Scheible (London: University of Minnesota Press), 309–328. Available at: https://www.taylorarnold.net/distantviewing/papers/2021-depth-deep-learning.pdf

[B6] Arora A. Barrett M. Lee E. Oborn E. Prince K. (2023). Risk and the future of AI: algorithmic bias, data colonialism, and marginalization. Inform. Organiz. 33:100478. 10.1016/j.infoandorg.2023.100478

[B7] Baumgartner R. (2021). Precision medicine and digital phenotyping: digital medicine's way from more data to better health. Big Data Soc. 8:20539517211066452. 10.1177/2053951721106645230953518

[B8] Bayliss K. (2022). Can england's national health system reforms overcome the neoliberal legacy? Int. J. Health Serv. 52, 480–491. 10.1177/00207314221115945 35892154 PMC9449431

[B9] Beer D. (2018). The Data Gaze: Capitalism, Power and Perception. London: Sage Research Methods.

[B10] Beer D. (2022). The problem of researching a recursive society: algorithms, data coils and the looping of the social. Big Data Soc. 9:20539517221104997. 10.1177/20539517221104997

[B11] Benech I. Wilson A. Dowell A. C. (1996). Evidence-based practice in primary care: past, present and future. J. Eval. Clin. Pract. 2, 249–263. 10.1111/j.1365-2753.1996.tb00055.x 9238598

[B12] Bennett S. (2023). “Transmuting values in artificial intelligence: investigating the motivations and contextual constraints shaping the ethics of artificial intelligence practitioners,” in Proceedings of the 2023 AAAI/ACM Conference on AI, Ethics, and Society (Montreal, QC).

[B13] Bennett S. Catanzariti B. Tollon F. (2025). “Everybody knows what a pothole is” representations of work and intelligence in AI practice and governance,” in AI & Society (London: Springer).

[B14] Bennett S. Claisse C. Luger E. Durrant A. C. (2023). “Unpicking epistemic injustices in digital health: on the implications of designing data-driven technologies for the management of long-term conditions,” in Proceedings of the 2023 AAAI/ACM Conference on AI, Ethics, and Society (Montreal, QC), 322–332.

[B15] Bhakuni H. Abimbola S. (2021). Epistemic injustice in academic global health. Lancet Global Health 9, e1465–1470. 10.1016/S2214-109X(21)00301-6 34384536

[B16] Byju A. (2024). “Godfather of AI” Predicted I Wouldn't Have a Job. He Was Wrong. New York City: The New Republic.

[B17] Campolo A. Schwerzmann K. (2023). From rules to examples: machine learning's type of authority. Big Data Soc. 10:20539517231188725. 10.1177/20539517231188725

[B18] Cass H. (2024). The cass review-implications and reassurance for practitioners. Child Adolesc. Ment. Health 29, 311–313. 10.1111/camh.12723 39155362

[B19] Chandhiramowuli S. Taylor A. S. Heitlinger S. Wang D. (2024). Making data work count. Proc. ACM on Human-Comp. Interact. 8, 1–26. 10.1145/3637367

[B20] Chapman R. (2023). Empire of Normality: Neurodiversity and Capitalism. London: Pluto Press.

[B21] Chapman R. Carel H. (2022). Neurodiversity, epistemic injustice, and the good human life. J. Soc. Philos. 53, 614–631. 10.1111/josp.12456

[B22] Couldry N. Mejias U. A. (2019). Data colonialism: rethinking big data's relation to the contemporary subject. Tele. New Media 20, 336–349. 10.1177/1527476418796632

[B23] Devlin H. Lunn J. (1986). The truth about the nhs? Br. Med. J. 292:1623. 10.1136/bmj.292.6536.1622 3087548 PMC1340700

[B24] Djulbegovic B. Guyatt G. H. Ashcroft R. E. (2009). Epistemologic inquiries in evidence-based medicine. Cancer Cont. 16, 158–168. 10.1177/107327480901600208 19337202

[B25] Domínguez Hernández A. Krishna S. Perini A. M. Katell M. Bennett S. Borda A.. (2024). “Mapping the individual, social and biospheric impacts of foundation models,” in The 2024 ACM Conference on Fairness, Accountability, and Transparency (Rio De Janeiro), 776–796.

[B26] Elish M. C. Boyd D. (2018). Situating methods in the magic of big data and AI. Commun. Monogr. 85, 57–80. 10.1080/03637751.2017.1375130

[B27] Ellis C. Jacobs M. Kendall D. (2021). The impact of racism, power, privilege, and positionality on communication sciences and disorders research: time to reconceptualize and seek a pathway to equity. Am. J. Speech-Lang. Pathol. 30, 2032–2039. 10.1044/2021_AJSLP-20-00346 34019772

[B28] Engle R. L. Mohr D. C. Holmes S. K. Seibert M. N. Afable M. Leyson J.. (2021). Evidence-based practice and patient-centered care: doing both well. Health Care Manage. Rev. 46, 174–184. 10.1097/HMR.0000000000000254 31233424 PMC8162222

[B29] Esayas S. Y. (2017). The idea of “emergent properties' in data privacy: towards a holistic approach. Int. J. Law Inform. Technol. 25, 139–178. 10.1093/ijlit/eaw015

[B30] Essén A. Lindblad S. (2013). Innovation as emergence in healthcare: unpacking change from within. Soc. Sci. Med. 93, 203–211. 10.1016/j.socscimed.2012.08.035 23021848

[B31] Fricker M. (2007). Epistemic Injustice: Power and the Ethics of Knowing. Oxford: Oxford University Press.

[B32] Goldenberg M. J. (2009). Iconoclast or creed?: objectivism, pragmatism, and the hierarchy of evidence. Perspect. Biol. Med. 52, 168–187. 10.1353/pbm.0.0080 19395818

[B33] Goldstein R. (2024). Epistemic injustice in the medical context: Introduction to special issue. Social Epistemol. 2024, 1–6. 10.1080/02691728.2024.2400096

[B34] Greenhalgh T. Snow R. Ryan S. Rees S. Salisbury H. (2015). Six biases' against patients and carers in evidence-based medicine. BMC Med. 13, 1–11. 10.1186/s12916-015-0437-x 26324223 PMC4556220

[B35] Grote T. Berens P. (2023). Uncertainty, evidence, and the integration of machine learning into medical practice. J. Med. Philos. 48, 84–97. 10.1093/jmp/jhac034 36630292

[B36] Guersenzvaig A. (2024). Can machine learning make naturalism about health truly naturalistic? A reflection on a data-driven concept of health. Ethics Inform. Technol. 26:2. 10.1007/s10676-023-09734-6

[B37] Gutin I. (2018). In BMI we trust: reframing the body mass index as a measure of health. Soc. Theory Health. 16:256. 10.1057/s41285-017-0055-0 31007613 PMC6469873

[B38] Guy M. McHale J. (2023). The health and care act 2022: new legislation-new legacy? Northern Ireland Legal Quart. 74, 657–663. 10.53386/nilq.v74i4.1136

[B39] Harrison P. (2022). The health and care act 2022. Gastrointest. Nurs. 20, 42–42. 10.12968/gasn.2022.20.6.42

[B40] Harrison S. Checkland K. (2009). “Evidenced-based practice in UK health policy,” in The New Sociology of the Health Service (London: Routledge), 131–152.

[B41] Harrison S. Wood B. (1999). Designing health service organization in the UK, 1968 to 1998: from blueprint to bright idea and “manipulated emergence”. Public Adm. 77, 751–768. 10.1111/1467-9299.00178

[B42] Hashem Y. Esnaashari S. Morgan D. Francis J. Poletaev A. Enock F. E.. (2024). One in Four UK Doctors are Using Artificial Intelligence: Exploring Doctors' Perspectives on AI After the Emergence of Large Language Models. London: Alan Turing Institute.

[B43] Heggen K. M. Berg H. (2021). Epistemic injustice in the age of evidence-based practice: The case of fibromyalgia. Human. Soc. Sci. Commun. 8, 1–6. 10.1057/s41599-021-00918-3

[B44] Higgins J. Thompson S. Deeks J. Altman D. (2002). Statistical heterogeneity in systematic reviews of clinical trials: a critical appraisal of guidelines and practice. J. Health Serv. Res. Policy 7, 51–61. 10.1258/1355819021927674 11822262

[B45] Hintz A. (2024). “AI, big data and bias: governing datafication through a data justice lens,” in Handbook of Media and Communication Governance (Cheltenham: Edward Elgar Publishing), 526–537.

[B46] Horgan D. Romao M. Morré S. A. Kalra D. (2020). Artificial intelligence: power for civilisation-and for better healthcare. Public Health Genom. 22, 145–161. 10.1159/000504785 31838476

[B47] Horton C. (2024). The cass review: Cis-supremacy in the UK's approach to healthcare for trans children. Int. J. Transgend. Health 2024, 1–25. 10.1080/26895269.2024.2328249 PMC12573551 41180936

[B48] Hunt J. (2013). A Mandate from the Government to NHS England: April 2014 to March 2015. Department of Health. London: Department of Health and Social Care.

[B49] Hussain-Gambles M. Atkin K. Leese B. (2004). Why ethnic minority groups are under-represented in clinical trials: a review of the literature. Health Soc. Care Commun. 12, 382–388. 10.1111/j.1365-2524.2004.00507.x 15373816

[B50] Jacobsen B. N. (2023). Machine learning and the politics of synthetic data. Big Data Soc. 10:20539517221145372. 10.1177/20539517221145372

[B51] Johnson S. (2002). Emergence: The Connected Lives of Ants, Brains, Cities, and Software. New York, NY: Simon and Schuster.

[B52] Joyce P. (2001). Governmentality and risk: setting priorities in the new NHS. Sociol. Health Illness 23, 594–614. 10.1111/1467-9566.00267

[B53] Jutel A. (2009). Sociology of diagnosis: a preliminary review. Sociol. Health Illness 31, 278–299. 10.1111/j.1467-9566.2008.01152.x 19220801

[B54] Klein R. (1982). Health care in the age of disillusionment. Br. Med. J. 1982, 2–4.

[B55] Klein R. (1983). The Politics of the National Health Service. Inverness: Longman.

[B56] Klingbeil A. Grützner C. Schreck P. (2024). Trust and reliance on AI-an experimental study on the extent and costs of overreliance on AI. Comput. Human Behav. 160:108352. 10.1016/j.chb.2024.108352

[B57] Leonelli S. Tempini N. (2020). Data Journeys in the Sciences. Cham: Springer Nature.

[B58] Miceli M. Posada J. Yang T. (2022). Studying up machine learning data: Why talk about bias when we mean power? Proc. ACM on Human-Comp. Interact. 6, 1–14. 10.1145/3492853

[B59] Miceli M. Schuessler M. Yang T. (2020). Between subjectivity and imposition: power dynamics in data annotation for computer vision. Proc. ACM on Human-Comp. Interact. 4, 1–25. 10.1145/3415186

[B60] Michaels J. A. (2021). Potential for epistemic injustice in evidence-based healthcare policy and guidance. J. Med. Ethics 47, 417–422. 10.1136/medethics-2020-106171 32461243

[B61] Milan S. Treré E. (2020). The rise of the data poor: the covid-19 pandemic seen from the margins. Soc. Media+ Soc. 6:l3. 10.1177/2056305120948233 34192035 PMC7424616

[B62] Moore J. (2019). AI for not bad. Front. Big Data 2:32. 10.3389/fdata.2019.00032 33693355 PMC7931857

[B63] Mullen E. J. Streiner D. L. (2006). “The evidence for and against evidence-based practice,” in Foundations of Evidence-Based Social Work Practice, 21–34.

[B64] Müskens J. L. Kool R. B. van Dulmen S. A. Westert G. P. (2022). Overuse of diagnostic testing in healthcare: a systematic review. BMJ Qual. Safety 31, 54–63. 10.1136/bmjqs-2020-012576 33972387 PMC8685650

[B65] Nelson A. D. (2019). Diagnostic dissonance and negotiations of biomedicalisation: Mental health practitioners'resistance to the DSM technology and diagnostic standardisation. Sociology of health & illness 41, 933–949. 10.1111/1467-9566.12876 30834559

[B66] Nettleton S. (2020). The Sociology of Health and Illness. Hoboken: John Wiley & Sons.

[B67] O'Connor D. (2015). “Emergent properties,” in Old and New Questions in Physics, Cosmology, Philosophy, and Theoretical Biology: Essays in Honor of Wolfgang Yourgrau (Cham: Springer), 719–732.

[B68] Ost K. Blalock C. Fagan M. Sweeney K. M. Miller-Hoover S. R. (2020). Aligning organizational culture and infrastructure to support evidence-based practice. Crit. Care Nurse 40, 59–63. 10.4037/ccn2020963 32476025

[B69] Perez C. C. (2019). Invisible Women: Data Bias in a World Designed for Men. New York: Abrams.

[B70] Piccialli F. Di Somma V. Giampaolo F. Cuomo S. Fortino G. (2021). A survey on deep learning in medicine: why, how and when? Inform. Fusion 66, 111–137. 10.1016/j.inffus.2020.09.006

[B71] Rath K. C. Khang A. Rath S. K. Satapathy N. Satapathy S. K. Kar S. (2024). “Artificial intelligence (AI)-enabled technology in medicine-advancing holistic healthcare monitoring and control systems,” in Computer Vision and AI-Integrated IoT Technologies in the Medical Ecosystem (Boca Raton, FL: CRC Press), 87–108.

[B72] Ratnani I. Fatima S. Abid M. M. Surani Z. Surani S. Fatima S. (2023). Evidence-based medicine: history, review, criticisms, and pitfalls. Cureus 15:2. 10.7759/cureus.35266 36968905 PMC10035760

[B73] Rennie O. (2024). Navigating the uncommon: challenges in applying evidence-based medicine to rare diseases and the prospects of artificial intelligence solutions. Med. Health Care Philos. 2024, 1–16. 10.1007/s11019-024-10206-x 38722452

[B74] Ricaurte P. (2022). Ethics for the majority world: AI and the question of violence at scale. Media, Cult. Soc. 44, 726–745. 10.1177/01634437221099612

[B75] Roberts M. Driggs D. Thorpe M. Gilbey J. Yeung M. Ursprung S.. (2021). Common pitfalls and recommendations for using machine learning to detect and prognosticate for covid-19 using chest radiographs and CT scans. Nat. Mach. Intellig. 3, 199–217. 10.1038/s42256-021-00307-0

[B76] Rogers L. O. Heard-Garris N. (2023). Documenting racial disparities or disrupting racism?: a call to center systems of power, privilege, and oppression in psychological and pediatric research. JAMA Pediatr. 177, 113–114. 10.1001/jamapediatrics.2022.3862 36574233

[B77] Roppelt J. S. Kanbach D. K. Kraus S. (2023). Artificial intelligence in healthcare institutions: a systematic literature review on influencing factors. Technol. Soc. 76:102443. 10.1016/j.techsoc.2023.102443

[B78] Rose D. Kalathil J. (2019). Power, privilege and knowledge: the untenable promise of co-production in mental “health”. Front. Sociol. 4:435866. 10.3389/fsoc.2019.00057 33869380 PMC8022626

[B79] Rose N. (1991). Governing by numbers: figuring out democracy. Accounti. Organiz. Soc. 16, 673–692. 10.1016/0361-3682(91)90019-B

[B80] Ross C. Swetlitz I. (2018). IBM's Watson supercomputer recommended “unsafe and incorrect” cancer treatments, internal documents show. Stat 25, 1–10. Available at: https://www.statnews.com/2018/07/25/ibm-watson-recommended-unsafe-incorrect-treatments/

[B81] Sackett D. L. Rosenberg W. M. Gray J. M. Haynes R. B. Richardson W. S. (1996). Evidence based medicine: what it is and what it isn't. BMJ 312:71–72. 10.1136/bmj.312.7023.71 8555924 PMC2349778

[B82] Stark L. (2023). Artificial intelligence and the conjectural sciences. BJHS Themes 8, 35–49. 10.1017/bjt.2023.3

[B83] Sturgeon D. (2014). The business of the nhs: the rise and rise of consumer culture and commodification in the provision of healthcare services. Critical Soc. Policy 34, 405–416. 10.1177/0261018314527717

[B84] Táíwò O. (2020). Being-in-the-room privilege: Elite capture and epistemic deference. Philosopher 108, 61–70. Available at: https://www.thephilosopher1923.org/post/being-in-the-room-privilege-elite-capture-and-epistemic-deference

[B85] Teig I. L. Baeroe K. Melberg A. Carlsen B. (2023). Governance determinants of health: exploring the structural impact of politicalization, bureaucratization and medical standardization on health inequity. Int. J. Health Govern. 28, 342–356. 10.1108/IJHG-03-2023-0031

[B86] Thomer A. K. Akmon D. York J. J. Tyler A. R. Polasek F. Lafia S.. (2022). The craft and coordination of data curation: Complicating workflow views of data science. Proc. ACM on Human-Comp. Interact. 6, 1–29. 10.1145/3555139

[B87] Topol E. (2019a). Deep Medicine: How Artificial Intelligence Can Make Healthcare Human Again. Edinburgh: Hachette UK.

[B88] Topol E. J. (2019b). High-performance medicine: the convergence of human and artificial intelligence. Nat. Med. 25, 44–56. 10.1038/s41591-018-0300-7 30617339

[B89] Uddin M. Darabidarabkhani Y. Shah A. Memon J. (2015). Evaluating power efficient algorithms for efficiency and carbon emissions in cloud data centers: a review. Renewable Sustain. Energy Rev. 51, 1553–1563. 10.1016/j.rser.2015.07.061

[B90] Wallace E. Fahey T. (2018). Use of tests in UK primary care. BMJ 363:k4895. 10.1136/bmj.k4895 30487294

[B91] Warden J. (1987). Patients rule ok? Br. Med. J. 294:1240. 10.1136/bmj.294.6581.1240 20742835 PMC1246412

[B92] Williams A. (1996). Qalys and ethics: a health economist's perspective. Social sci. Med. 43, 1795–1804. 10.1016/S0277-9536(96)00082-2 8961422

[B93] Zahlan A. Ranjan R. P. Hayes D. (2023). Artificial intelligence innovation in healthcare: literature review, exploratory analysis, and future research. Technol. Soc. 74:102321. 10.1016/j.techsoc.2023.102321

[B94] Zając H. D. Avlona N. R. Kensing F. Andersen T. O. Shklovski I. (2023). “Ground truth or dare: Factors affecting the creation of medical datasets for training AI,” in Proceedings of the 2023 AAAI/ACM Conference on AI, Ethics, and Society (Montreal, QC), 351–362.

